# Effect of occupational exposure to vat-textile dyes on follicular and luteal hormones in female dye workers in Abeokuta, Nigeria

**DOI:** 10.4314/ahs.v24i1.17

**Published:** 2024-03

**Authors:** Oluwatosin O Soyinka, Akinwunmi F Akinsanya, Festus A Odeyemi, Adebayo A Amballi, Kolawole S Oritogun, Omobola A Ogundahunsi

**Affiliations:** 1 Department of Chemical Pathology and Immunology, College of Health Sciences, Sagamu Campus, Olabisi Onabanjo University, Ago Iwoye, Nigeria; 2 Department of Obstetrics and Gynaecology, College of Health Sciences, Sagamu Campus, Olabisi Onabanjo University, Ago Iwoye, Nigeria; 3 Department of Community Medicine, College of Health Sciences, Sagamu Campus, Olabisi Onabanjo University, Ago Iwoye, Nigeria

**Keywords:** Follicular phase, luteal phase, dye workers, sex hormone, premenopausal

## Abstract

**Background:**

Some synthetic dyes used mainly in textile industries have been associated with endocrine disruption, resulting in infertility, among other disorders. It is unknown if occupational exposure to Vat textile dyes among premenopausal dyers alters hormonal levels.

**Objectives:**

We aimed at determining the probable effects of occupational exposure to Vat dyes on reproductive hormones of female textile dyers in the follicular and luteal phases while relating this to age categories and duration of exposure.

**Methods:**

Thirty-three premenopausal Vat textile dyers at “Itoku”, Abeokuta, Nigeria, among a population of about 80 female dyers were age and sex-matched with 55 non-exposed (control) female participants. Using semi-structured questionnaires, socio-demographic, occupational details and the LMP of participants were obtained. Serum samples were collected in follicular and luteal phases and assayed for female sex hormones using Enzyme Immunoassay. Mann-Whitney U and Z- statistic were used for comparison of the two groups. P-value < 0.05 was considered to be significant.

**Results:**

In the follicular phase, the result showed a lower mean FSH ranking (in age category ≤20 years) and higher (p<0.05) Estradiol ranking (in age category 31-40 years) in the exposed than the unexposed. Mean ranks of Progesterone and Estradiol in the luteal phase (age category 31-40 years) were higher (p<0.05) in the exposed, while Estradiol (age category ≥41years) ranked lower (p<0.05). Prolactin demonstrated a significant inverse relationship with the duration of exposure.

**Conclusion:**

Occupational exposure to Vat dye among female dyers in Abeokuta is associated with some sex hormone disruption which appears to be age and duration of exposure-related.

## Introduction

The local textile dyeing in Abeokuta, South West of Nigeria, otherwise known as “Adire and Kampala” making, has been practised from time immemorial as an indigenous and economically viable business that serves as an essential hub for economic engagement and a credible source of local and possibly foreign exchange earnings. The business essentially involves the use of Vat dyes; these are anthraquinone and indigo-based dyes with carbonyl functional group^1^. Other countries known for the production or utilization of vat dyes include China and Pakistan 2,3 In actual fact textile industry play a key role in the economy of most countries 3.

A good proportion of the workforce is considerably formed by the growing number of women of reproductive age. Mounting concerns abound on the negative impact of variable and continuous quantum of exposure to Vat dyes among these women, particularly those of reproductive age. Similarly, the absence of data on this peculiar mode of indigenous textile dyeing among the locals appears compelling.

In his study of Knowledge, Attitude and Practices, among dye workers in Abeokuta, Akintayo^4^. reported that workers spend an average of nine hours per day and eleven months per year, and only a few of the workers use personal protective equipment such as fit for the skin, thus showing the likelihood of exposure to the adverse effects of these Vat dyes.

Some textile dyes have been shown to be harmful to female reproductive health through the mechanism of endocrine disruption 5. Female textile dye exposure has been associated with infertility 6. Similarly, the same was observed in male mice 7. Furthermore, lac dye, a food dye, was also reported to demonstrate adverse reproductive effects in mice 8. Hair dye was another type of dye with such a report. Nagata et al. 9 observed an association between the long-term use of hair dye and the increase in fasting testosterone levels in premenopausal women. In the same population, higher free and total Estradiol levels were marginally and significantly associated with a greater frequency of applying non-permanent hair dyes.

A decade before this, however, in their review, Nohynek et al. 10 did not report any adverse human reproductive effects in consumer or occupational hair dye exposure. Reproductive hormonal imbalance is a common cause of female infertility, a social problem affecting 10 to 15% of married couples 11. Among this population of dye workers, the prevalence of infertility was reported to be 40.5% 12. It is unknown if there are altered hormone levels among premenopausal women engaging in dye work in Abeokuta, Nigeria. Also, hormonal levels can be affected by age and have been related to the duration of exposure to chemicals or occupations 13,14. Furthermore, it can be studied in both the follicular and luteal phases.

This study was therefore designed to determine the possible effects of Vat dyes on the reproductive hormone profile of textile workers of various age categories in the follicular and luteal phases and to evaluate the association of the effects with duration of exposure

## Materials and methods

### Study design

This study took place at “Itoku” market, Abeokuta, Southwest, Nigeria (as earlier described by Soyinka *et al.*^15^. Using convenient sampling technique, the total population of female vat textile dye workers was considered, at the time of the study, only 80 female indigenous dyers were registered with the association of textile dyers in this small-scale industry. A meeting was held with the dyers where the aim and objectives of the study were described.

It was a cohort study and retrospective in nature, as the dyers had been exposed before the start of this study. They were 33 female Vat textile dye workers who served as the exposed group. They were age and sex-matched with 55 non-exposed female participants who served as the control group. Exposed subjects that were included in the study were non-pregnant, non-lactating, and within reproductive age (between 16 and 49years), and had not, in the past three (3) months breastfed. Those included among the exposed group were dyers with two years' minimum duration of exposure within the dyeing community. Excluded from the study were amenorrheic subjects, contraceptive users, those who could not be traced to the location of dyeing, those who were not available for blood sampling, and textile dyers who only worked with indigo dyes and not anthraquinone-based dyes.

The participants were classified into four different age groups: ≤20 years (or early reproductive age), 21-30 (mid reproductive age), 31-40 (late reproductive age) and ≥41 years (perimenopausal age). Ethical approval was received from the Scientific and Ethical Review Committee of Olabisi Onabanjo University Teaching Hospital, Sagamu, Ogun State.

### The control group

The control group consisted of 55 female participants who had no prior or current exposure to textile dyes either through occupational or non-occupationally means. They were mostly hospital attendants from the State Hospital, “Ijaiye”, Abeokuta and “Egba Medical Centre”, “Isabo”, Abeokuta. Others included Staff of a group of schools, also in Abeokuta.

### Data collection

Tools used for data collection included the following:

**Questionnaires:** The socio-demographic and occupational details were captured using an interviewer-administered questionnaire.

**Bathroom scale and meter rule:** bathroom scale (Hanson, China) and meter rule were used to measure weight (Kg) and height (cm) respectively as described by Soyinka *et al.*^12^. Body mass index was calculated from body weight in (Kg) divided by height (m) squared. BMI=weight (Kg)/height (m^2^).

### Blood sampling and techniques

Two sets of blood samples were collected from the participants. Sampling time was arrived at by using a small handy calendar given to each dye worker to report her last menstrual period (LMP, 1st day of bleeding during a particular menstrual period). The follicular phase blood sample was collected between the 5^th^ and 10^th^ day of the menstrual cycle, while that of the luteal was collected between the 19^th^ and 21^st^ day. The specific day of collection was dependent on agreement between the principal Investigator and the dye worker. Detail of menstrual cycle, regularity and others have earlier been reported 15.

The blood samples were allowed to clot and centrifuged at 700 x g for 5 minutes i. The serum samples were separated from the red cells, and each subject's sample was put in three aliquots and transported in ice bags to the former Centre for Research in Reproductive (CRRH), Sagamu, Ogun State, Nigeria, where they were stored at -20°C before the various analyses at the same centre.

### Biochemical assays

Biochemical assays were performed by Enzyme Immunoassay (EIA) methods using kits from Immunometrics, United Kingdom. The procedures for the assays were carried out according to the instructions in the manual in the kits. The optical density (OD) of the samples were read on Serozyme 1 from Serono Diagnostic USA.

### Statistical analyses

The Statistical Package for the Social Sciences (SPSS), version 16.0, was initially used for data input and analysis; however, version 20 was utilized for the final analyses. Z- statistics was used to compare the means between two groups of continuous variables for normally distributed data. Non-parametric analyses were used where the data were observed to be skewed (not normally distributed): Mann-Whitney U for comparison of two independent groups (the output being mean rank), and Spearman rank correlation was used to determine relationships between quantitative variables. Results were expressed as means ± (SD) for unskewed and mean rank for skewed data. P values < 0.05 were considered significant.

## Results

The mean age and the BMI of the female dye workers were 28.48±8.39 (ranging between 16 and 49 years) and 23.95±5.00 (17.58 and 42.22), while that of the exposed were 31.33 ± 8.79 (ranging between 17 and 48 years) and 25.28 ± 4.55 (16.44 and 34.37), respectively. There were no significant differences P>0.05 between the two groups.

The mean job duration of the exposed was 10.29±8.23 (ranging between 2 and 50 years).

The population distribution was asymmetrical, hence non-parametric test was applied. Comparisons between the mean ranks of the exposed and the unexposed group are shown in table 2 above. Only FSH was found to be significantly lower in the exposed group. (P<0.05).

**Table 2:** Serum hormones in the follicular phase of menstrual cycle of the female vat dye exposed workers and the unexposed

Sex Hormones	Mean Rank		Z-value	P-value
	Exposed	Unexposed		
LH (IU/L)	49.20	41.68	1.34	0.180
FSH (IU/L)	34.30	50.62	2.91	0.004*
Prolactin (mIU/L)	44.65	44.53	0.01	0.992
Progesterone nmol/L	51.06	40.56	1.65	0.099
Estradiol nmol/L	49.70	40.52	1.87	0.061

LH= Luteinizing hormone, FSH =Follicle Stimulating hormone. *Statistically significant value; p<0.05 is significant, P= probability, Z = test statistics.

As shown in Table 3 above is a comparison of the mean rank of the exposed and the unexposed group. No significant differences in the mean ranks of the two groups.

**Table 3:** Serum hormones in the luteal phase of menstrual cycle of vat dye exposed workers and the unexposed

Sex Hormone	Mean Rank		Z-value	P-value
	**Exposed**	**Unexposed**		
LH (IU/L)	48.05	30.25	1.60	0.11
FSH (IU/L)	37.48	47.06	1.73	0.08
Prolactin (mIU/L)	42.06	45.13	0.55	0.58
Progesterone nmol/L	47.64	41.88	0.11	0.91
Estradiol nmol/L	43.11	43.73	0.22	0.82

LH= luteinizing hormone, FSH=Follicle stimulating hormone; Results are expressed as mean rank; n=number of subjects used; P= probability; Z= test statistics

## Discussion

Human exposure to chemicals has been documented to affect female hormone function 16,17. These chemicals, via occupational or environmental exposure, act as reproductive toxins 17, 18, causing reproductive dysfunction through disruption of the hormonal balance, which is important for proper functioning 16, 19. Disruption of the hypothalamic-pituitary-gonadal axis via altered secretion of hypothalamic GnRH, for the control of gonadotropins (LH and FSH) pulsatile secretion from the anterior pituitary has been documented 20. Changes with the adverse reproductive consequence in the gonadal steroids' metabolism via cytochrome P450 gene families have been linked with chemicals acting as reproductive toxins 20. As a result, ovulation disturbance and thus fertility disorders occur when there is hormonal imbalance (HI) in females 16,21. Several textile dyes have been reported to be potential endocrine disruptors 5, 22. This study, therefore, was set up to examine the likely effects of occupational exposure to Vat dyes on the reproductive hormone profile of female textile dye workers of various age categories in both follicular and luteal phases of their menstrual cycles, since hormonal level varies within the menstrual cycle and exposures may cause a change in levels at a certain time of the menstrual cycle 23,24,25. This was done by comparing hormonal levels in the dyers with age and sex-matched unexposed participants. The relationship between levels of reproductive hormone and duration of exposure to Vat dyes was also determined 26.

In the follicular phase of the menstrual cycle of women exposed to textile dyes, we observed alteration only in the level of FSH. This reduced level, could be a result of reduced synthesis in the anterior pituitary gland, which is modulated by a gonadotropin-releasing hormone from the hypothalamus. This could not be ascertained because we did not measure the levels of GnRH. Another likely reason for a lower level of FSH was negative feedback from the target organ (Ovary). This is unlikely, as the levels of Estradiol and Progesterone (products of the ovary) remain unchanged in this phase. Our result is similar to that of Zemlianova 27. He reported a lower level of FSH among female dye workers. FSH stimulates the development of ovarian follicles in females. With significantly low FSH, as observed in this study, there is a likelihood of impairment in follicular development, which could lead to ovulation failure and infertility, as reported by Ragaa *et al.* 22 in their study of effects of reactive dyes on Nile Tilapia (*Oreochromis Niloticus*). Soyinka *et al.* 12, however, did not report a significant increased prevalence of infertility among these textile dyers compared with the control.

This study extended to looking at the effects of age on the hormonal levels of women occupationally exposed to textile dyes. When the women were categorized into various age categories, as shown in Table 4 it was observed that the reduction in the level of FSH was at the lowest age category (≤20) years. It might be said that this was not sufficiently reduced to affect the target hormones because no alteration was seen in the Estradiol as well as the Progesterone. Furthermore, we observed an increase in the Estradiol level in the age category 31-40 years. The same was seen in the luteal phase, as shown in Table 5 and in this same age category. In addition, Progesterone level also increased. It can therefore be said that some sex hormones fluctuate in the female dye workers and effects differ with respect to age category. However, by the perimenopausal age (≥41), Estradiol became reduced. According to Horstman *et al.* 28, Estradiol level decreases with age; In this case, this reduction could not have been due to age because we utilized a case-control method, and the reduction was only observed in the exposed participants.

**Table 4:** Comparison of the mean rank of the follicular hormone levels of the dye exposed workers with the control in various age categories

Age category (years)	Sex Hormones	Mean Rank		Z-value	P-value
		**Exposed**	**Unexposed**		
**≤20**		(n=8)	(n=8)		
	LH (IU/L)	9.31	7.69	0.69	0.49
	FSH (IU/L)	5.50	11.50	2.53	0.01*
	Prolactin (mIU/L)	9.94	7.06	1.23	0.22
	Progesterone nmol/L	9.44	7.56	0.80	0.43
	Estradiol nmol/L	7.56	9.44	0.79	0.43
**21-30**		n=13	n=19		
	LH (IU/L)	17.38	15.89	0.44	0.66
	FSH (IU/L)	13.42	18.61	1.54	0.12
	Prolactin (mIU/L)	15.54	17.16	0.49	0.93
	Progesterone nmol/L	19.38	14.53	1.44	0.15
	Estradiol nmol/L	19.23	13.67	1.68	0.54
**31-40**		n=10	n=18		
	LH (IU/L)	15.85	13.75	0.65	0.52
	FSH (IU/L)	12.75	15.47	0.84	0.40
	Prolactin (mIU/L)	16.15	13.58	0.80	0.43
	Progesterone nmol/L	13.85	14.86	0.31	0.76
	Estradiol nmol/L	19.20	11.89	2.25	0.02*
**≥41**		n=2	n=12		
	LH (IU/L)	7.00	6.40	0.22	0.83
	FSH (IU/L)	7.50	6.30	0.43	0.67
	Prolactin (mIU/L)	2.25	7.35	1.86	0.06
	Progesterone nmol/L	10.75	5.65	1.84	0.07
	Estradiol nmol/L	5.00	6.80	0.65	0.52

LH= luteinizing hormone, FSH =Follicle stimulating hormone; n =number of subjects used; p= probability; Z-test statistics; *Statistically significant value; P≤0.05 is significant.

**Table 5:** Luteal phase hormone levels of the female vat dye exposed workers and the unexposed in various age categories

Age category (Years)	Sex Hormones	Mean Rank		Z-value	P-value
		Exposed	Unexposed		
**≤20**		(n=8)	(n=8)		
	LH (IU/L)	7.00	8.88	0.81	0.42
	FSH (IU/L)	6.07	9.69	1.57	0.12
	Prolactin (mIU/L)	9.71	6.50	1.39	0.17
	Progesterone nmol/L	8.21	7.81	0.17	0.86
	Estradiol nmol/L	6.86	9.00	0.93	0.36
**21-30**		n=14	n=19		
	LH (IU/L)	17.04	16.13	0.23	0.79
	FSH (IU/L)	15.15	17.42	0.67	0.50
	Prolactin (mIU/L)	16.15	16.74	0.17	0.86
	Progesterone nmol/L	16.11	17.66	0.19	0.85
	Estradiol nmol/L	16.12	16.76	0.54	0.59
**31-40**		n=10	n=18		
	LH (IU/L)	16.00	11.31	1.55	0.12
	FSH (IU/L)	11.61	14.50	0.92	0.35
	Prolactin (mIU/L)	11.94	15.03	0.96	0.34
	Progesterone nmol/L	19.44	11.28	2.27	0.02*
	Estradiol nmol/L	19.67	11.17	2.52	0.01*
**≥41**		n=2	n=10		
	LH (IU/L)	11.00	5.50	1.59	0.11
	FSH (IU/L)	6.50	6.50	0.00	1.00
	Prolactin (mIU/L)	2.25	7.35	1.86	0.06
	Progesterone nmol/L	2.50	7.30	1.72	0.09
	Estradiol nmol/L	1.50	7.50	2.15	0.03*

LH= luteinizing hormone, FSH =Follicle stimulating hormone; n =number of subjects used; p= probability; Z-test statistics; *Statistically significant value; P<0.05 is significant

In relating sex hormone level to the duration of exposure, as indicated in Table 6 we noted that out of all the sex hormones, only the level of Prolactin was significantly reduced over time. This occurs in both the follicular and the luteal phases of the menstrual cycle of the women. Reduced Prolactin levels may therefore be a sensitive indicator of the hazardous effect of occupational exposure to Vat dye exposure, particularly among women of reproductive age. This is because hormone imbalance is associated with reproductive dysfunctions 29. Our observation agrees with the suggestion that Prolactin may be a sensitive indicator of early effects in toxicological research 30. Beyond this, it will be good to test for the effects of a longer duration of exposure on prolactin levels. Prolactin has been reported to respond in different ways to chemical exposures; this is related to different neurotransmitters involved in modulating its secretion. According to Lafuente *et al.* 31, factors affecting levels of Prolactin in such exposures include doses. In occupational exposures, the longer the duration, the more the likelihood of an increased quantum of exposure.

**Table 6:** Correlation studies of length of time in occupation and sex hormone levels in female dye-exposed population

	Sex Hormone	R	P-value
**Female follicular** **(n=20)**			
	LH	0.05	0.77
	FSH	0.12	0.51
	Prolactin	-0.45	0.01*
	Progesterone	-0.12	0.52
	Estradiol	0.09	0.63
**Female luteal (n=39)**			
	LH	0.11	0.55
	FSH	0.07	0.70
	Prolactin	-0.42	0.02*
	Progesterone	-0.35	0.05
	Estradiol	0.20	0.28

r= correlation coefficient; p= probability; * represents significant values at p≤ 0.05 LH =luteinizing hormone, FSH = Follicle stimulating hormone.

## Conclusion

Female Vat dye workers of early reproductive age (<20 years) in Abeokuta have considerably lower levels of FSH during the follicular phase of the menstrual cycle.

Estradiol levels increased in both the follicular and luteal phases in women of mid-reproductive age (31 to 40 years); an increase in the Progesterone levels was observed in the luteal phase. Additionally, it was shown that perimenopausal women (>41 years) had lower levels of estradiol. Prolactin was shown to be inversely related to the duration of exposure.

These unexpected observations are likely to be as a result of the exposure to the Vat dye on the hypothalamic- pituitary- gonadal axis. This serves as a scientific basis on the need for personal protective equipment in occupation exposure to Vat dyes.

## Figures and Tables

**Table 1:** Age, BMI and job duration of vat dye workers and the unexposed participants

Parameter	Exposed n=33	Unexposed n=55	Z	P-value
**Age**	28.48±8.39	31.33 ±8.79	1.49	0.136
**BMI**	23.95±5.00	25.28 ±4.55	1.21	0.226
**Job Duration**	10.29±8.23	NA.	NA.	NA

NA= Not applicable; BMI = Body Mass Index, Z= Z-test, P= P-value, n=sample no

## Data Availability

Dr. Oluwatosin Soyinka, one of the Authors, is in possession of the datasets used in this study. It can be made available upon reasonable request.

## References

[1] Walters A, Santillo D, Johnston P (2005). An Overview of Textiles Processing and Related Environmental Concerns.

[2] Li S, Shi Y, Huang H, Tong Y, Wu S, Wang Y (2022). Fermentation Blues: Analyzing the Microbiota of Traditional Indigo Vat Dyeing in Hunan, China. Microbiol Spectr.

[3] Younas T, Tayyaba N, Ayub A, Ali S (2021). Textile Fabric's and Dyes, Tekstilna Industrija. Broj.

[4] Akintayo WL (2013). Knowledge, attitude and practice on the use of personal protective equipment by traditional resist fabrics workers in Abeokuta, Nigeria. Arabian J Bus Manag Review (Kuwait Chapter).

[5] Bazin I, Ibn Hadj Hassine A, Haj Hamouda Y, Mnif W, Bartegi A, Lopez-Ferber M, De Waard M, Gonzalez C (2012). Estrogenic and anti-estrogenic activity of 23 commercial textile dyes. Ecotoxicology and Environmental Safety Journal.

[6] Rachootin P, Olsen J (1983). The risk of infertility and delayed conception associated with exposures in the Danish workplace. Journal of occupational medicine.: official publication of the Industrial Medical Association.

[7] Fernandes FH, Bustos-Obregon E, Salvadori DM (2015). Disperse Red 1 (textile dye) induces cytotoxic and genotoxic effects in mouse germ cells. Reproductive toxicology (Elmsford, NY).

[8] Tanaka T (1997). Reproductive and neurobehavioural effects of lac dye administered in the diet to mice. Food Additives and Contaminants.

[9] Nagata C, Wada K, Tsuji M, Hayashi M, Takeda N, Yasuda K (2015). Association of hair dye use with circulating levels of sex hormones in premenopausal Japanese women. European journal of public health.

[10] Nohynek GJ, Fautz R, Benech-Kieffer F, Toutain H (2005). Toxicity and human health risk of hair dyes. Food and chemical toxicology. an international journal published for the British Industrial Biological Research Association.

[11] Ekpe EL, Osuji KC, Ejikem Eke CM (2020). Pattern of Hormonal Imbalance among Women of Child –bearing age in a Tertiary Healthcare Centre in Southern Nigeria. Research Journal of Obstetrics and Gynecology.

[12] Soyinka OO, Oritogun KS, Amballi AA, Olorundami BO, Ogundahunsi OA (2020). Effects of Occupational Exposure to Vat Dye on Fertility and Pregnancy Outcome of Women Dye Users. Archives of Basic and Applied Medicine.

[13] Eldesouki MA, Sharaf NE, Abdel Shakour AA, Mohamed MS, Hussein AS, Hasani IW (2013). Study of the Effect of Occupational Exposure to Volatile Organic Compounds (VOC's) on Male Reproductive Hormones. World Journal of Medical Sciences.

[14] Yang Y, Lu XS, Li DL, Yu YJ (2013). Effects of environmental lead pollution on blood lead and sex hormone levels among occupationally exposed group in an E-waste dismantling area. Biomedical and environmental sciences. BES.

[15] Soyinka OO, Ogundahunsi OA, Oritogun KS, Daniel OJ, Olorundami BO (2017). Age at Menarche and Menstrual Pattern of Vat Textile Dye-exposed Workers at Itoku, Abeokuta, Southwest, Nigeria. African Journal of Science & Nature.

[16] Bretveld RW, Thomas CM, Scheepers PT, Zielhuis GA, Roeleveld N (2006). Pesticide exposure: the hormonal function of the female reproductive system disrupted?. Reproductive biology and endocrinology: RB&E.

[17] Van Duursen MBM, Boberg J, Christiansen S, Connolly L, Damdimopoulou P, Filis P, Fowler PA, Gadella BM, Holte J, Jääger K, Johansson HKL, Li T, Mazaud-Guittot S, Parent AS, Salumets A, Soto AM, Svingen T, Velthut-Meikas A, Bay Wedebye E, Xie Y, Van den Berg M (2020). Safeguarding Female Reproductive Health Against Endocrine Disrupting Chemicals—The FREIA Project. Int J Mol Sci.

[18] Luderer U, Hieb M, Diaz J, Baker B, American College of Occupational and Environmental Medicine (2011). Reproductive and developmental hazard management guidance: ACOEM Task Force on Reproductive Toxicology. Journal of occupational and environmental medicine.

[19] Scsukova S, Rollerova E, Bujnakova Mlynarcikova A (2016). Impact of endocrine disrupting chemicals on onset and development of female reproductive disorders and hormone-related cancer. Reproductive biology.

[20] Thurston SW, Ryan L, Christiani DC, Snow R, Carlson J, You L, Cui S, Ma G, Wang L, Huang Y, Xu X (2000). Petrochemical exposure and menstrual disturbances. American journal of industrial medicine.

[21] Sharma S, Khinchi MP, Sharma N, Agrawal D, Gupta MK (2011). Female Infertility: An Overview. International Journal of Pharmaceutical Sciences and Research.

[22] Ragaa FF, Abdel-Rahim EA, Shallan MA, Wagdy KBK, Samah MB, Fagr KHA (2021). Assessment of Holothuria Atra Extracts Against Synthetic Textile Dyes Induced Endocrine Disorders, Biochemical and Genetic Alterations in Nile Tilapia (Oreochromis Niloticus ). Inter J pharm Res.

[23] Ekpenyong CE, Davies K, Daniel N (2013). Effects of Gasoline Inhalation on Menstrual Characteristics and the Hormonal Profile of Female Petrol Pump Workers. Journal of Environmental Protection.

[24] Pollack AZ, Schisterman EF, Goldman LR, Mumford SL, Albert PS, Jones RL, Wactawski-Wende J (2011). Cadmium, lead, and mercury in relation to reproductive hormones and anovulation in premenopausal women. Environmental health perspectives.

[25] Ranga Rao TV, Guruswamy T, Khawas BH (2003). Occupational Health Status of Workers Exposed to Female Hormones in a Pharmaceutical Plant. Indian Journal of Occupational and Environmental Medicine.

[26] Udeogu CH, Ogbu ISI, Mbachu AN, Ugwu MC, Odo OF, Ugwu O (2020). Sex Hormones Levels in Male Welders in Nnewi, South-Eastern Nigeria. Asian Journal of Biochemistry, Genetics and Molecular Biology.

[27] Zemlianova MA (2014). Evaluating cytogenetic and hormonal state in female workers of the dyeboard industry of fabric preparation. Meditsina Trudai Promyshlennaia Ekologiia.

[28] Horstman AM, Dillon EL, Urban RJ, Sheffield-Moore M (2012). The role of androgens and estrogens on healthy aging and longevity. J Gerontol A Biol Sci Med Sci.

[29] Faiza H, Duodpota MR, Shaikh F, Rafiq M, Murtaza G, Naqvi SHA (2021). Association of Hormonal Imbalance, Life-style and Clinical Attributes with Female Infertility. JPUMHS.

[30] de Burbure C, Buchet JP, Leroyer A, Nisse C, Haguenoer JM, Mutti A, Smerhovsky Z, Cikrt M, Trzcinka-Ochocka M, Razniewska G, Jakubowski M, Bernard A (2006). Renal and neurologic effects of cadmium, lead, mercury, and arsenic in children: evidence of early effects and multiple interactions at environmental exposure levels. Environmental health perspectives.

[31] Lafuente A, Cano P, Esquifino A (2003). Are cadmium effects on plasma gonadotropins, Prolactin, ACTH, GH and TSH levels, dose-dependent?. Biometals: an international journal on the role of metal ions in biology, biochemistry, and medicine.

